# Detection and distribution of two dominant alleles associated with the sweet kernel phenotype in almond cultivated germplasm

**DOI:** 10.3389/fpls.2023.1171195

**Published:** 2023-04-14

**Authors:** Concetta Lotti, Anna Paola Minervini, Chiara Delvento, Pasquale Losciale, Liliana Gaeta, Raquel Sánchez-Pérez, Luigi Ricciardi, Stefano Pavan

**Affiliations:** ^1^ Department of Agriculture, Food, Natural Resources and Engineering, University of Foggia, Foggia, Italy; ^2^ Department of Soil, Plant and Food Sciences, Section of Plant Genetics and Breeding, University of Bari Aldo Moro, Bari, Italy; ^3^ Council for Agricultural Research and Economics-Research Centre for Agriculture and Environment (CREA-AA), Bari, Italy; ^4^ Plant Breeding Department, Fruit Breeding Group, CEBAS-CSIC, Campus Universitario de Espinardo, Espinardo, Spain

**Keywords:** almond, kernel taste, allele mining, marker-assisted selection, breeding

## Abstract

Almond [*Prunus dulcis* Miller (D. A. Webb), syn. *Prunus amygdalus* L.)] is the major tree nut crop worldwide in terms of production and cultivated area. Almond domestication was enabled by the selection of individuals bearing sweet kernels, which do not accumulate high levels of the toxic cyanogenic glucoside amygdalin. Previously, we showed that the *Sweet kernel* (*Sk*) gene, controlling the kernel taste in almond, encodes a basic helix loop helix (bHLH) transcription factor regulating the amygdalin biosynthetic pathway. In addition, we characterized a dominant allele of this gene, further referred to as *Sk-1*, which originates from a C^1036^→T missense mutation and confers the sweet kernel phenotype. Here we provide evidence indicating that the allele further referred to as *Sk-2*, originally detected in the cultivar “Atocha” and arising from a T^989^→G missense mutation, is also dominantly inherited and confers the sweet kernel phenotype in almond cultivated germplasm. The use of single nucleotide polymorphism (SNP) data from genotyping by sequencing (GBS) for population structure and hierarchical clustering analyses indicated that *Sk-2* occurs in a group of related genotypes, including the widespread cultivar “Texas”, descending from the same ancestral population. KASP and dual label functional markers were developed for the accurate and high-throughput selection of the *Sk-1* and *Sk-2* alleles, and the genotyping of a panel of 134 almond cultivars. Overall, our results provide further insights on the understanding of the almond cultivation history. In addition, molecular marker assays and genotypic data presented in this study are expected to be of major interest for the conduction of almond breeding programs, which often need to select sweet kernel individuals in segregant populations.

## 1 Introduction

Almond [*Prunus dulcis* Miller (D.A. Webb), syn. *Prunus amygdalus* L.] is the tree nut species with the highest cultivated area and production worldwide (FAOSTAT data, 2021). U.S. accounts for about 80% of the almond global yield (1.7 million tonnes of shelled product), followed by Australia (6%), and Spain (5%) (FAOSTAT data, 2021; Pérez de los Cobos et al., 2021).

The kernels of wild almond relatives are bitter and highly toxic to humans, as they accumulate the cyanogenic glucoside amygdalin. Almond domestication, which took place in the Fertile Crescent about 12,000 years ago, was therefore signed by the selection of sweet kernel mutants (Sánchez-Pérez et al., 2019). Genetic studies indicated that the kernel taste is controlled by a single gene located on the almond chromosome 5, referred to as *Sweet kernel* (*Sk*) (Dicenta and García, 1993; Sánchez-Pérez et al., 2010). More recently, we showed that the *Sk* gene encodes a basic helix-loop-helix (bHLH) transcription factor. In addition, we characterized a dominant allele of this gene, further referred to as *Sk-1*, conferring the sweet kernel phenotype and originating from a C^1036^→T substitution in the gene coding sequence. This leads to a nonsynonymous change (Leu^346^→Phe) in the protein helix loop helix (HLH) domain which impairs the expression of the first two genes of the amygdalin biosynthetic pathway, encoding the P450 cytochromes PdCYP79D16 and PdCYP71AN24 (Thodberg et al., 2018; Sánchez-Pérez et al., 2019).

Resequencing of the *Sk* gene in a panel of 30 sweet kernel cultivars showed the occurrence of the *Sk-1* allele in all cases but one, represented by the Spanish cultivar “Atocha”. However, “Atocha” displayed, at the heterozygous state, another nucleotide substitution (T^989^→G), also leading to a nonsynonymous change (Leu^330^→Arg) in the HLH domain (Sánchez-Pérez et al., 2019).

Almond breeding programs often involve the cross between cultivars heterozygous for the wild-type recessive *sk* allele. This implies that bitter kernel individuals must be discarded in segregating progenies. Phenotypic selection for kernel taste is costly and time consuming, as the almond juvenile phase prevents the evaluation of fruit traits prior to 3-4 years. Therefore, several studies were addressed to the identification of DNA markers suitable for assisted selection (Sánchez-Pérez et al., 2007; Sánchez-Pérez et al., 2010; Ricciardi et al., 2018).

Restriction fragment length polymorphism (RFLP), simple sequence repeat (SSR), and cleaved amplified length polymorphism (CAPS) molecular markers, located at variable genetic distance from the *Sk* locus, are currently available for the selection of sweet kernel genotypes (Sánchez-Pérez et al., 2007; Sánchez-Pérez et al., 2010; Ricciardi et al., 2018). Although they can be obtained with relatively inexpensive laboratory equipment, they are not ideal for large scale genotyping, as they require restriction enzyme digestion and/or manual scoring of electrophoretic profiles. In contrast, the Kompetitive Allele Specific PCR (KASP) technology, developed at LGC Genomics and based on competitive allele-specific PCR amplification of target sequences and endpoint fluorescence (Smith and Maughan, 2015), provides an automatized, high-throughput and cost-effective solution for genotyping and marker-assisted selection (Semagn et al., 2014; Nadeem et al., 2018; Ayalew et al., 2019; Li et al., 2022). In addition, dual label assays, based on fluorescently tagged and allele specific probes, are also suitable for high-throughput genotyping and used in plant breeding (Broccanello et al., 2018).

Functional or diagnostic markers are defined as those markers designed on the DNA polymorphism affecting phenotypic variation (Andersen and Lübberstedt, 2003; Ma et al., 2017). Functional markers are in complete linkage with functional motifs, and thus are fully predictive of the phenotype; in addition, they overcome issues related to other marker types, such as the lack of polymorphism between the parental genotypes of breeding populations, and the need of phenotypic validation of selected individuals (Pavan et al., 2013; Salgotra and Stewart, 2020). Thus, functional markers were indicated as major contributors to the development of precision plant breeding (Salgotra and Stewart, 2020).

Here, we performed allele mining on the *Sk* gene, indicating that two different alleles associated with the almond sweet kernel phenotype were selected with domestication and occur in cultivated germplasm. We characterized the distribution of the two *Sk* alleles in a large almond germplasm collection, including cultivars worldwide used in breeding programs. Finally, we developed KASP and dual label functional markers targeting the *Sk* DNA polymorphisms.

## 2 Materials and methods

### 2.1 Plant material and genomic DNA isolation

Plant material considered in this study was sampled from the *ex situ* collections of CREA-AA (Council for Agricultural Research and Analysis of Agricultural Economics—Research Centre for Agriculture and Environment), Bari, Italy, and CEBAS-CSIC (Spanish National Research Council—Center for Edaphology and Applied Biology of the Segura River), Murcia, Spain. In total, 144 individuals were considered, including 134 sweet kernel cultivars originating from main almond-growing countries (**Supplementary Table 1**) and 10 bitter kernel individuals selected from the segregant “R1000” x “Desmayo Largueta” segregant population previously described by Ricciardi et al. (2018) and Sánchez-Pérez et al. (2019). Genomic DNA was extracted by young leaf material, using the DNeasy Plant Mini Kit (Qiagen, Hilden, Germany) according to the manufacturer’s protocol. DNA quality and concentration were checked using 0.8% agarose gel electrophoresis and the NanoDrop 1000 spectrophotometer (ThermoScientific, Pittsburgh, PA, U.S.).

### 2.2 Marker development

Molecular markers assays were designed on the C^1036^T and the T^989^G single nucleotide polymorphisms (SNPs) occurring within the coding sequence of the *Sk* gene, based on the KASP and the dual label technologies. For KASP assays, three primers were designed on the sequence flanking the SNP position: two allele-specific forward primers, marked with the FAM and HEX fluorescence dyes, and a common reverse primer (**Table 1**). PCR reactions were performed at LGC genomics Service Lab (Hoddesdon, U.K.) according to standard protocols. Output fluorescence result tables were converted into scatter plots using the ggplot2 R package (Wickham, 2016).

**Table 1:** Information on KASP assays designed in this study to detect the *Sk-1* and *Sk-2* alleles.

Allele	Target SNP	Allele specific primer 1	Allele specific primer 2	Common primer
*Sk-1*	T/C	GATGTATGCCACAGCATCTGCAAA	ATGTATGCCACAGCATCTGCAAG	AATGTGTCGAAGATGGACAGATCTTCTTT
*Sk-2*	T/G	CACATTCGGAACAACAGAGCGGA	ACATTCGGAACAACAGAGCGGC	CGAGAGAAGCTTAACCATCGCTTCTA

The ddPCR mutation detection assay, adapted to the CFX 96 Real-Time PCR detecting system (Bio-Rad Laboratories, Hercules, CA, U.S.) was used as dual label strategy. Sequences extending at both sides of the SNP position for 61 base pairs were used to design primers and probes, whose sequences were not provided by the supplier. PCR mixes were prepared in 10 µl reaction containing 1x SsoAdvanced Universal Probes Supermix, 1x target (FAM) and wild-type (HEX) primers/probes, and about 50 ng of genomic DNA. PCR reactions were carried with the following conditions: 95° C for 10 min, 40 cycles at 94° C for 30 s followed by 58°C for 30s. Genotypic calls were obtained through the allelic discrimination function available in the Bio-Rad CFX Manager v.3.1. software. Fluorescence result tables were exported and converted into scatter plots using the ggplot2 R package (Wickham, 2016).

Validation of genotypic calls on 23 selected individuals was performed by the inspection of electropherograms resulting from Sanger sequencing. This was performed at Eurofins Genomics (Ebersberg, Germany) using the primer combination SkFw (5’-GGTGCTTGAACTGGCTTCTC-3’)/SkRev (5’-CTTCCGATCCCAAAATCT-3’), flanking the *Sk* coding sequence (NCBI accession number MK041092). In addition, the occurrence of the T^989^→G mutation in the cultivar “Texas” was confirmed blasting the *Sk* coding sequence against the “Texas” reference genome sequence (Alioto et al., 2020).

### 2.3 Search of genomic variation data and quality control

The raw variant call format (VCF) SNP file reported by (Pavan et al., 2021) (doi: 10.6084/m9.figshare.12205652), which refers to the application of genotyping by sequencing (GBS) (Elshire et al., 2011) on an almond diversity panel, was used. Data relative to the cultivars genotyped in this study for the *Sk* locus were extracted. Then, quality control was performed by filtering for biallelic SNP loci with minor allele frequency >0.05 and missing rate <0.3, and for cultivars with missing data <0.4, using Tassel 5 (Bradbury et al., 2007). Finally, SNPs were pruned for linkage disequilibrium using the flag indep-pairwise 50 5 0.5 available in PLINK v.1.90 (Purcell et al., 2007).

### 2.4 Population structure analysis and hierarchical clustering

The analysis of genetic structure was performed by the software ADMIXTURE (Alexander et al., 2009), assuming from 1 to 10 ancestral populations and running 1,000 bootstrap replicates to estimate parameter standard errors. The most suitable number of ancestral populations was identified in correspondence with the lowest cross‐validation (CV) error. In accordance with the previous study by Pavan et al. (2021), individuals were assigned to one of the ancestral populations when their membership coefficient (qi) for that population was higher than 0.6, otherwise they were considered of admixed ancestry. Hierarchical clustering was performed using the AWclust R package (Gao and Starmer, 2008), based on the allele-sharing distance and the Ward’s minimum variance clustering algorithm.

## 3 Results

### 3.1 Development of KASP and dual label functional markers for the selection of the *Sk-1* allele

**Figure 1:**
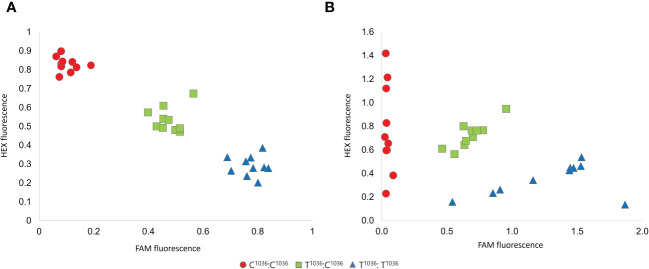
Validation of the KASP **(A)** and dual label **(B)** assays designed on the *Sk-1* allele. Red circles, green squares and blue triangles indicate individuals belonging to the genotypic clusters associated with zero (C^1036^:C^1036^), one (T^1036^:C^1036^) or two (T^1036^T^1036^) *Sk-1* copies, respectively.

Two KASP and dual label assays were designed to detect the *Sk-1* allele, originating from the C^1036^→T mutation in the gene coding sequence (Sánchez-Pérez et al., 2019). When tested on a panel of 30 individuals, each of the two assays yielded three fluorescence clusters (**Figure 1**). In addition, genotypic calls resulting from the two assays were fully consistent. As expected, the *Sk-1* allele was not detected in ten bitter kernel individuals selected from a previously described F_1_ segregant population (Ricciardi et al., 2018), which were therefore assigned to the C^1036^:C^1036^ genotypic cluster. Sweet kernel cultivars were assigned to two genotypic clusters, predicted to have one (T^1036^:C ^1036^) or two (T^1036^:T^1036^) copies of the *Sk-1* allele. With no exception, Sanger sequencing confirmed the correct call of the T^1036^:C ^1036^ and T^1036^:T^1036^ genotypic clusters.

Once performed assay validation, we genotyped additional 114 sweet kernel cultivars, originating from the main almond-growing countries. Overall, the KASP and dual label assays yielded a call rate of 97% and 99.2%, respectively. All the genotypic calls, reported in **Supplementary Table 1**, were consistent between the two assays.

### 3.2 The *Sk-2* allele is also associated with the almond sweet kernel phenotype

Although at least one copy of the *Sk-1* allele was detected in most cultivars, exceptions were found, represented by the cultivars “Texas”, “Pizzuta d’Avola”, and “Mollar de Tarragona” (**Supplementary Table 1**). Sanger sequencing confirmed the C^1036^:C^1036^ genotype of these cultivars.

Sánchez-Pérez et al. (2019) reported, for the *Sk* gene of the Spanish cultivar “Atocha”, a C^1036^:C^1036^ genotype and, at the same time, a heterozygous point mutation (T^989^→G) leading to a nonsynonymous change (Leu^330^→Arg) in the HLH protein domain. Notably, according to Sanger sequencing, the C^1036^:C^1036^ cultivars also carried at least one copy of the G^989^ mutation. Indeed, “Texas” and “Pizzuta D’Avola” were scored as heterozygous (T^989^:G^989^), whereas “Mollar de Tarragona” was scored as homozygous (G^989^:G^989^). The occurrence of the T^989^→G mutation in “Texas” was further confirmed by BLAST search against the newly released reference genome of the same cultivar (Alioto et al., 2020).

**Figure 2:**
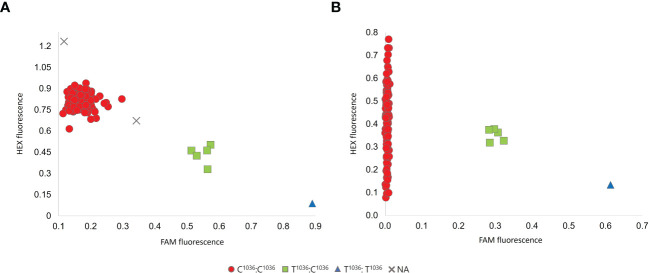
Results of the KASP **(A)** and dual label **(B)** assays designed on the *Sk-2* allele. Red circles, green squares and blue triangles indicate individuals belonging to the genotypic clusters associated with zero (T^989^:T^989^), one (T^989^:G^989^) or two (G^989^:G^989^) *Sk-2* copies, respectively. Grey crosses indicate individuals associated with missing genotypic call.

The results above mentioned indicated that, besides *Sk-1*, another dominant allele conferring the sweet kernel phenotype, arising from a T^989^→G mutation, was selected with domestication and occurs within almond cultivated germplasm. We further named this allele *Sk-2* and designed new KASP and dual label assays for its specific detection, which were tested on the same panel of cultivars above mentioned. As shown in **Figure 2**, both assays resulted in three fluorescence clusters. The call rates were 98.5% and 100% for the KASP and the dual label assay, respectively. In addition, genotypic calls were again fully consistent. “Mollar de Tarragona” was genotyped as homozygous for the *Sk-2* allele (G^989^:G^989^), whereas “Texas” and “Pizzuta D’Avola” were genotyped as heterozygous (T^989^:G^989^), in accordance with the results of Sanger Sequencing. In addition, the cultivars “Nikitsky”, “Butte” and “Del Cid” were also genotyped as heterozygous (**Supplementary Table 1**). Overall, the relative frequencies of the *Sk-1* and *Sk-2* alleles in the panel of almond cultivars subjected to genotyping were 96.5% and 3.5%, respectively.

### 3.3 Genetic relationships among cultivars carrying the two *Sk* alleles

Aiming to investigate the origin of the two *Sk* alleles, we exploited the availability of DNA polymorphism data for most of the cultivars genotyped in this study, including “Mollar de Tarragona”, “Nikisky”, “Texas” and “Pizzuta D’Avola”, carrying the *Sk-2* allele (Pavan et al., 2021). The application of quality control procedure resulted in a dataset of 114 cultivars and 44,638 SNPs.

**Figure 3:**
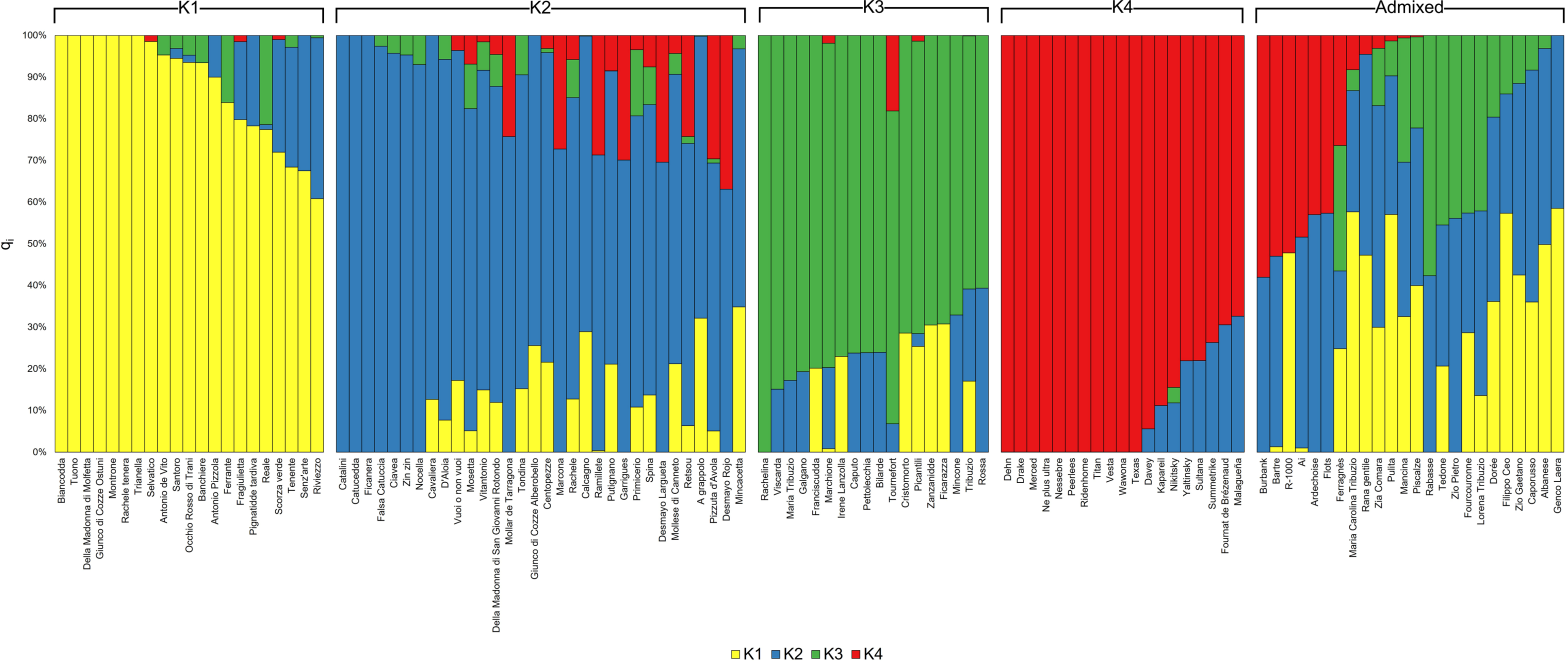
Results of ADMIXTURE population structure analysis for a model with four ancestral populations, named from K1 to K4. Each cultivar is represented by a vertical bar. In turn, each bar is divided into segments whose length and color represent the proportion of the genome (qi) contributed by each ancestral population. Cultivars assigned to one of the ancestral populations have a membership coefficient (qi) for that population >0.6. The remaining cultivars are assigned to the admixed group.

**Figure 4:**
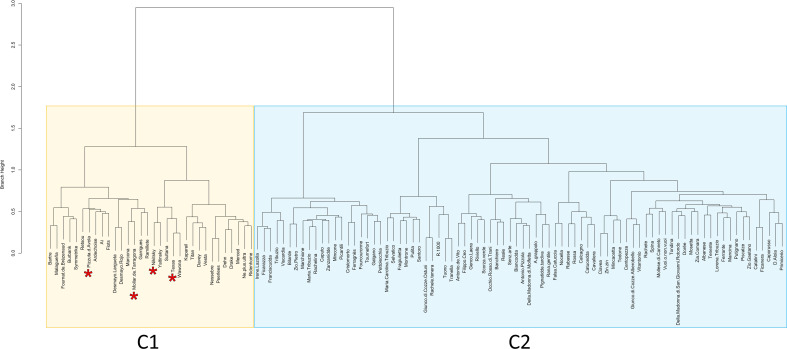
Results of hierarchical clustering with the AWclust algorithm. The two main clusters of the dendrogram (C1 and C2) are shaded with different colors. The names of the cultivars “Mollar de Tarragona”, “Nikisky”, “Texas” and “Pizzuta D’Avola”, harboring at least one copy of the *Sk-2* allele, are highlighted with an asterisk.

Population structure analysis indicated a model with four ancestral populations (named from K1 to K4) as suitable to explain data (**Supplementary Figure 1**). With a few exceptions, cultivars assigned to the ancestral populations K1, K2 and K3 originated from Italy, whereas cultivars assigned to the ancestral population K4 originated from U.S., except for the Ukranian cultivar “Nikitsky” (**Figure 3**; **Table 2**). Concerning the four cultivars in the panel bearing the *Sk-2* allele, two of them (“Texas” and “Nikitsky”) were assigned to K4, and two (“Mollar de Tarragona” and “Pizzuta D’Avola) to the admixed group. The inspection of membership coefficients (qi) returned by the ADMIXTURE parametric model, indicating the estimated percentage of the genome deriving from each ancestry, revealed that the K4 ancestry was the only one associated with all the four cultivars bearing *Sk-2* (**Table 2**), suggesting that the *Sk-2* mutation arose or was selected in the ancestral population K4. Hierarchical clustering resulted in the identification of two main clusters (named C1 and C2), with C1 including the four cultivars mentioned above carrying the *Sk-2* allele (**Figure 4**).

**Table 2:** ADMIXTURE membership coefficient for the four ancestral populations K1, K2, K3 and K4, relative to four cultivars (“Mollar de Tarragona”, “Nikisky”, “Texas” and “Pizzuta D’Avola”) carrying at least one copy of the *Sk-2* allele.

Cultivar	K1	K2	K3	K4
Mollar de Tarragona	0.0	0.8	0.0	0.2
Nikitsky	0.0	0.1	0.0	0.8
Pizzuta d’Avola	0.1	0.6	0.0	0.3
Texas	0.0	0.0	0.0	1.0

## 4 Discussion

We recently reported the isolation of the almond *Sk* gene and the identification of its dominant C^1036^→T mutant allele, re-named here as *Sk-1*, which confers the sweet kernel phenotype (Sánchez-Pérez et al., 2019). In this study, allele mining indicated that a second *Sk* mutant allele, originating from a T^989^→G mutation and named *Sk-2*, also occurs in almond cultivated germplasm. The presence of at least one copy of *Sk-2* in all the sweet kernel cultivars lacking *Sk-1* indicates that *Sk-2* is also a dominant allele functionally related to the sweet kernel phenotype. In addition, it suggests that *Sk-1*-and *Sk-2* are the only mutations leading to the sweet kernel taste that occur in almond cultivated germplasm. Both the *Sk-1* and *Sk-2* mutations lead to nonsynonymous changes in the protein HLH domain, which is known to play a major role in the dimerization of bHLH transcription factors (Murre, 2019).

Sweet kernel cultivars exhibit trace amounts of amygdalin, and variation in amygdalin concentration in sweet kernel cultivars are of commercial importance, as they have an influence on the kernel taste (Arrázola et al., 2012; Lee et al., 2013). Thus, it would be interesting to investigate whether the different genotypic combinations harboring the *Sk-1* and *Sk-2* alleles are associated with significantly different amygdalin concentrations.

The lower frequency of *Sk-2* with respect to *Sk-1* in almond cultivated germplasm, together with the occurrence of *Sk-2* in specific genetic groups (**Figures 3**, **4**), suggest that *Sk-1* was the first allele to be selected with domestication, and that the *Sk-2* mutation arose or was selected in a later stage during the almond cultivation history. Population structure analysis identified the ancestral population K4, significantly contributing to the differentiation of Spanish, French, and U.S. cultivars, as the one in which *Sk-2* first appeared. Importantly, although literature indicates that U.S. almond breeding originates from the introduction of French germplasm in late XIX century, genotyping failed to detect the *Sk-2* allele, occurring in the U.S. cultivars “Texas” and “Butte”, in French germplasm. Conversely, the occurrence of *Sk-2* in several Spanish cultivars (“Del Cid”, “Atocha”, “Mollar de Tarragona”) suggests that at least part of the U.S. almond germplasm might originate from Spanish introductions.

The identification of molecular markers suitable for the selection of sweet kernel individuals has been for a long time one of the main research aims for almond breeding. Differently from the molecular markers reported so far, which are located at various genetic distances from the *Sk* gene (Sánchez-Pérez et al., 2007; Sánchez-Pérez et al., 2010; Ricciardi et al., 2018), those identified in this study directly target *Sk* polymorphisms causing kernel taste variation, and thus fall within the definition of functional markers provided by Andersen and Lübberstedt (2003). Functional markers are considered optimal to assist selection, as they overcome issues related to recombination and allow precision breeding (Lau et al., 2015). Both KASP and dual label assays used in this study represent state-of-the-art and high-throughput technologies for marker-assisted selection, which are widely used in plant breeding (Broccanello et al., 2018; Radanović et al., 2022). KASP technology is known to provide a lower cost per data point, however it provides a less efficient separation of genotypic clusters and requires higher amount of DNA template (Ayalew et al., 2019). In our study, KASP assays resulted in slightly lower call rates than dual label assays.

In conclusion, we provide evidence indicating that two mutations of the same transcription factors were selected with domestication to make almonds palatable. In addition, we describe functional marker assays allowing the accurate and high-throughput selection of sweet kernel individuals in almond breeding programs. Finally, we report genotypic data of a large germplasm collection, including cosmopolitan cultivars and the four most widely used founding clones for almond breeding programs worldwide, i.e. “Cristomorto”, “Tuono”, “Nonpareil” and “Texas” (Pérez de los Cobos et al., 2021), which are expected to be of great relevance for the almond breeding community.

## Data availability statement

The original contributions presented in the study are included in the article/**Supplementary Material**. Further inquiries can be directed to the corresponding author.

## Author contributions

SP conceived the study. LG, PL and RS-P assembled the genetic materials. LR and SP provided funds to the research. CL and AM performed lab work. CD and AM performed data analyses. SP wrote the manuscript draft. All authors contributed to the article and approved the submitted version. 
