# Supplementary material for: Detection and distribution of two dominant alleles associated with the sweet kernel phenotype in almond cultivated germplasm
Source: Front Plant Sci. 2023 Apr 14;14:1171195. doi: 10.3389/fpls.2023.1171195 (PMC10145170; doi:10.3389/fpls.2023.1171195)
Supplement: Supplementary file 1 [file DataSheet_1.docx]

Supplementary Material

Detection and distribution of two alleles of the *Sk* gene, controlling the kernel taste in almond [*Prunus dulcis* Miller (D.A. Webb)]

**Concetta Lotti, Anna Paola Minervini, Chiara Delvento, Pasquale Losciale, Liliana Gaeta, Raquel Sánchez-Pérez, Luigi Ricciardi, Pavan Stefano^*^**

***Correspondence:** Prof. Stefano Pavan: [stefano.pavan@uniba.it](mailto:stefano.pavan@uniba.it)

# Supplementary Figures and Tables

## Supplementary Figure


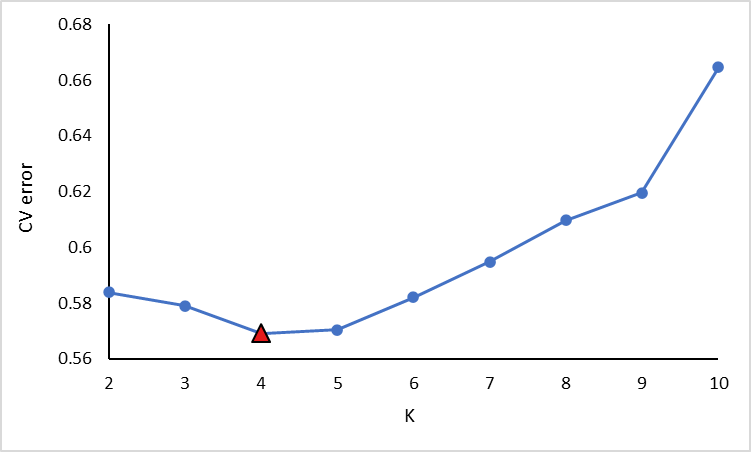
**Supplementary Figure 1.** ADMIXTURE cross-validation (CV) error estimates for a number of ancestral populations (K) ranging from 1 to 10. The red triangle indicates the lowest CV error detected for K=4.

## Supplementary Table

## Supplementary Table 1. Results from genotyping 134 almond cultivars with the marker assays developed in this study. T^1036^ and G^989^ are indicative of the *Sk-1* and *Sk-2* alleles, respectively. Genotypic calls from the KASP and dual label assays were fully consistent, thus they are reported as a single column for each nucleotide position. Genotyping of cultivars indicated in bold was confirmed by Sanger sequencing.

| **Cultivar** | **Genotype at position C1036T** | **Genotype at position T989G** | **Collection** | **Origin** |
| --- | --- | --- | --- | --- |
| A grappolo | TT | TT | CREA-AA | Italy |
| Ai | TT | TT | CREA-AA | France |
| Albanese | TC | TT | CREA-AA | Italy |
| Antonio de Vito | TT | TT | CREA-AA | Italy |
| Antonio Pizzola | TC | TT | CREA-AA | Italy |
| **Ardechoise** | TC | TT | CREA-AA | France |
| Banchiere | TC | TT | CREA-AA | Italy |
| Bartre | TT | TT | CREA-AA | Spain |
| **Biancodda** | TC | TT | CREA-AA | Italy |
| Bilarde | TT | TT | CREA-AA | Italy |
| Burbank | TC | TT | CREA-AA | U.S. |
| Butte | TC | TG | CEBAS-CSIC | U.S. |
| Calcagno | TC | TT | CREA-AA | Italy |
| Caporusso | TT | TT | CREA-AA | Italy |
| Caputo | TC | TT | CREA-AA | Italy |
| Catalini | TT | TT | CREA-AA | Italy |
| Catuccia | TC | TT | CREA-AA | Italy |
| Catucedda | TC | TT | CREA-AA | Italy |
| Cavaliera | TC | TT | CREA-AA | Italy |
| **Centopezze** | TT | TT | CREA-AA | Italy |
| Chino | TC | TT | CREA-AA | Italy |
| Ciavea | TT | TT | CREA-AA | Italy |
| Cosimo di Bari | TC | TT | CREA-AA | Italy |
| Crimsky | TC | TT | CREA-AA | Ukraine |
| **Cristomorto** | TT | TT | CREA-AA | Italy |
| D'Aloia | TT | TT | CREA-AA | Italy |
| Davey | TC | TT | CREA-AA | U.S. |
| Dehn | TC | TT | CREA-AA | U.S. |
| Del Cid | TC | TG | CEBAS-CSIC | Spain |
| Del lago | TT | TT | CREA-AA | Italy |
| Della Madonna di Molfetta | TC | TT | CREA-AA | Italy |
| Della Madonna di San Giovanni Rotondo | TC | TT | CREA-AA | Italy |
| **Desmayo Largueta** | TC | TT | CREA-AA | Spain |
| Desmayo Rojo | TC | TT | CREA-AA | Spain |
| Dorée | TC | TT | CREA-AA | France |
| Drake | TC | TT | CREA-AA | U.S. |
| Falsa Catuccia | TC | TT | CREA-AA | Italy |
| Ferragnès | TT | TT | CREA-AA | France |
| Ferrante | TC | TT | CREA-AA | Italy |
| Ficanera | TT | TT | CREA-AA | Italy |
| Ficarazza | TT | TT | CREA-AA | Italy |
| **Filippo Ceo** | TT | TT | CREA-AA | Italy |
| Flots | TT | TT | CREA-AA | France |
| Fourcouronne | TC | TT | CREA-AA | France |
| Fournat de Brézenaud | TT | TT | CREA-AA | France |
| Fragiulietta | TC | TT | CREA-AA | Italy |
| Fragiulio | TC | TT | CREA-AA | Italy |
| Franciscudda | TT | TT | CREA-AA | Italy |
| **Galgano** | TT | TT | CREA-AA | Italy |
| Garrigues | TC | TT | CEBAS-CSIC | Spain |
| Genco Laera | TC | TT | CREA-AA | Italy |
| Gioia | TT | TT | CREA-AA | Italy |
| Giunco di Cozze Alberobello | TT | TT | CREA-AA | Italy |
| Giunco di Cozze Ostuni | TT | TT | CREA-AA | Italy |
| Hasboreita | TT | TT | CEBAS-CSIC | Spain |
| Irene Lanzolla | TT | TT | CREA-AA | Italy |
| IXL | TC | TT | CREA-AA | U.S. |
| Jordanolo | TC | TT | CREA-AA | U.S. |
| Kapareil | TT | TT | CREA-AA | U.S. |
| **Lauranne** | TT | TT | CEBAS-CSIC | Spain |
| Lorena Tribuzio | TC | TT | CREA-AA | Italy |
| Malagueña | TT | TT | CREA-AA | Spain |
| Mancina | TC | TT | CREA-AA | Italy |
| Marchione | TT | TT | CREA-AA | Italy |
| Marcona | TC | TT | CREA-AA | Spain |
| Maria Carolina Tribuzio | TC | TT | CREA-AA | Italy |
| Maria Tribuzio | TT | TT | CREA-AA | Italy |
| **Merced** | TC | TT | CREA-AA | U.S. |
| Miagkoskorlupy | TC | TT | CREA-AA | Ukraine |
| Mincacetta | TT | TT | CREA-AA | Italy |
| Mincone | TT | TT | CREA-AA | Italy |
| **Mollar de Tarragona** | CC | GG | CREA-AA | Spain |
| Mollese di Canneto | TT | TT | CREA-AA | Italy |
| Montrone | TC | TT | CREA-AA | Italy |
| Mosetta | TT | TT | CREA-AA | Italy |
| Naturale di Montevalle | TT | TT | CREA-AA | Italy |
| **Ne plus ultra** | TC | TT | CREA-AA | U.S. |
| Nessebre | TC | TT | CREA-AA | Ukraine |
| Nikitsky | TC | TG | CREA-AA | Ukraine |
| Nocella | TT | TT | CREA-AA | Italy |
| **Nonpareil** | TC | TT | CREA-AA | U.S. |
| Occhio Rosso di Trani | TT | TT | CREA-AA | Italy |
| Pappamucco | TC | TT | CREA-AA | Italy |
| Peerless | TC | TT | CREA-AA | U.S. |
| Pettolecchia | TT | TT | CREA-AA | Italy |
| **Piangente** | TT | TT | CREA-AA | Italy |
| Picantili | TT | TT | CREA-AA | Ukraine |
| Pignatidde tardiva | TC | TT | CREA-AA | Italy |
| Piscalze | TC | TT | CREA-AA | Italy |
| **Pizzuta d'Avola** | CC | TG | CREA-AA | Italy |
| Primicerio | TT | TT | CREA-AA | Italy |
| Pulita | TC | TT | CREA-AA | Italy |
| Putignano | TC | TT | CREA-AA | Italy |
| R1000 | TC | TT | CEBAS-CSIC | France |
| **Rabasse** | TC | TT | CREA-AA | France |
| Rachele | TT | TT | CREA-AA | Italy |
| Rachele tenera | TT | TT | CREA-AA | Italy |
| Rachelina | TT | TT | CREA-AA | Italy |
| Ramillete | TT | TT | CEBAS-CSIC | Spain |
| Rana | TT | TT | CREA-AA | Italy |
| Rana gentile | TT | TT | CREA-AA | Italy |
| Reale | TT | TT | CREA-AA | Italy |
| Retsou | TT | TT | CREA-AA | Greece |
| Ridenhome | TC | TT | CREA-AA | U.S. |
| Riviezzo | TC | TT | CREA-AA | Italy |
| **Rossa** | TT | TT | CREA-AA | Italy |
| Santoro | TC | TT | CREA-AA | Italy |
| Scarpetta | TT | TT | CREA-AA | Italy |
| Scorza verde | TT | TT | CREA-AA | Italy |
| Senz'arte | TT | TT | CREA-AA | Italy |
| Spina | TT | TT | CREA-AA | Italy |
| Sultana | TC | TT | CREA-AA | France |
| Summetrike | TC | TT | CREA-AA | Greece |
| Tedone | TT | TT | CREA-AA | Italy |
| **Tenente** | TC | TT | CREA-AA | Italy |
| **Texas** | CC | TG | CREA-AA | U.S. |
| **Titan** | TT | TT | CEBAS-CSIC | U.S. |
| Tondina | TT | TT | CREA-AA | Italy |
| **Tournefort** | TT | TT | CREA-AA | France |
| Trianella | TC | TT | CREA-AA | Italy |
| Tribuzio | TC | TT | CREA-AA | Italy |
| Troito | TC | TT | CREA-AA | Italy |
| **Tuono** | TC | TT | CREA-AA | Italy |
| Vesta | TC | TT | CREA-AA | U.S. |
| Viscarda | TT | TT | CREA-AA | Italy |
| Vitantonio | TC | TT | CREA-AA | Italy |
| Vuoi o non vuoi | TT | TT | CREA-AA | Italy |
| Wawona | TT | TT | CEBAS-CSIC | U.S. |
| **Yaltinsky** | TC | TT | CREA-AA | Ukraine |
| Zanzanidde | TT | TT | CREA-AA | Italy |
| Zia Comara | TT | TT | CREA-AA | Italy |
| **Zin zin** | TT | TT | CREA-AA | Italy |
| Zio Gaetano | TT | TT | CREA-AA | Italy |
| Zio Pietro | TT | TT | CREA-AA | Italy |
